# 2-Amino-5-(4-carboxyl­atophen­yl)­pyridinium monohydrate

**DOI:** 10.1107/S1600536812035854

**Published:** 2012-08-23

**Authors:** Xiao-Hong Wei, Hong Yan, Wei-Gong Lin

**Affiliations:** a College of Mechanical and Materials Engineering, China Three Gorges University, Yichang 443002, People’s Republic of China

## Abstract

The title compound, C_12_H_10_N_2_O_2_·H_2_O, crystallizes as a zwitterion in which the pyridine N atom is protonated and the carb­oxy –OH group is deprotonated. The benzene and pyridinium rings are inclined with a dihedral angle of 6.63 (5)° between them. In the crystal, inter­molecular O—H⋯O and N—H⋯O hydrogen-bonding inter­actions link adjacent mol­ecules into a two-dimensional double layered supra­molecular network.

## Related literature

For the use of pyridine­carboxyl­ate acid in coordination chemistry and for related structures, see: Jia *et al.* (2007 ▶); Zhang *et al.* (2011 ▶).
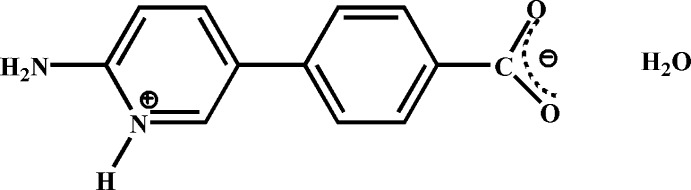


## Experimental

### 

#### Crystal data

C_12_H_10_N_2_O_2_·H_2_O
*M* *_r_* = 232.24
Monoclinic, 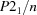

*a* = 7.796 (2) Å
*b* = 7.808 (2) Å
*c* = 18.480 (5) Å
β = 95.165 (14)°
*V* = 1120.4 (6) Å^3^
*Z* = 4
Mo *K*α radiation
μ = 0.10 mm^−1^
*T* = 296 K
0.23 × 0.16 × 0.15 mm

#### Data collection

Bruker SMART CCD diffractometer
Absorption correction: multi-scan (*SADABS*; Sheldrick, 1996 ▶) *T*_min_ = 0.977, *T*_max_ = 0.985
11449 measured reflections
2554 independent reflections
1505 reflections with *I* > 2σ(*I*)
*R*_int_ = 0.090

#### Refinement

*R*[*F* ^2^ > 2σ(*F* ^2^)] = 0.080
*wR*(*F* ^2^) = 0.208
*S* = 1.01
2554 reflections
160 parameters
3 restraints
H atoms treated by a mixture of independent and constrained refinement
Δρ_max_ = 0.17 e Å^−3^
Δρ_min_ = −0.16 e Å^−3^

### 

Data collection: *SMART* (Bruker, 1999 ▶); cell refinement: *SAINT* (Bruker, 1999 ▶); data reduction: *SAINT*; program(s) used to solve structure: *SHELXS97* (Sheldrick, 2008 ▶); program(s) used to refine structure: *SHELXL97* (Sheldrick, 2008 ▶); molecular graphics: *SHELXTL* (Sheldrick, 2008 ▶); software used to prepare material for publication: *SHELXTL*.

## Supplementary Material

Crystal structure: contains datablock(s) I, global. DOI: 10.1107/S1600536812035854/jj2150sup1.cif

Structure factors: contains datablock(s) I. DOI: 10.1107/S1600536812035854/jj2150Isup2.hkl

Supplementary material file. DOI: 10.1107/S1600536812035854/jj2150Isup3.cml

Additional supplementary materials:  crystallographic information; 3D view; checkCIF report

## Figures and Tables

**Table 1:** Hydrogen-bond geometry (Å, °)

*D*—H⋯*A*	*D*—H	H⋯*A*	*D*⋯*A*	*D*—H⋯*A*
N1—H1*A*⋯O1^i^	0.86	1.82	2.648 (3)	160
N2—H2*A*⋯O2^i^	0.86	2.07	2.911 (4)	167
N2—H2*B*⋯O3	0.86	2.09	2.921 (4)	163
O3—H3*C*⋯O2^ii^	0.86 (2)	1.99 (2)	2.849 (4)	178 (4)
O3—H3*D*⋯O1^iii^	0.86 (2)	1.91 (2)	2.768 (4)	176 (5)

Symmetry codes: (i) 
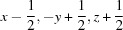
; (ii) 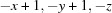; (iii) 
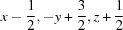
.

**Fig. 1.**
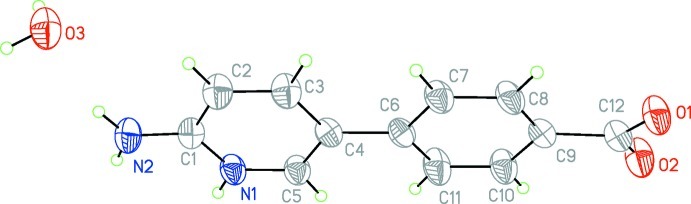
The structure of the title compound with the atom-numbering scheme showing displacement ellipsoids at the 30% probability level for non-H atoms.

**Fig. 2.**
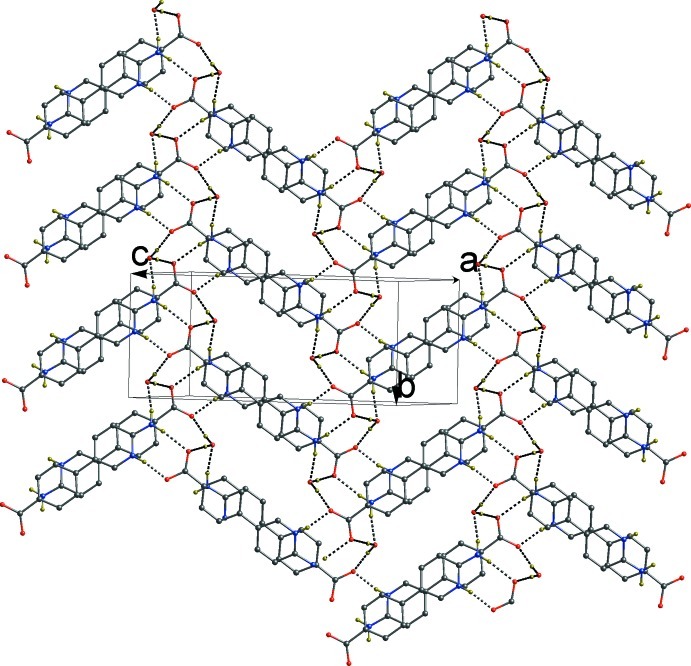
The two-dimensional supramolecular network formed by N—H···O and O—H···O (dashed lines) hydrogen-bonding interactions.

## supplementary crystallographic information

### Comment

Rigid pyridinecarboxylate ligands have been extensively employed to react with metal ions and generate coordination polymers with fascinating structures and properties(Jia *et al.*, 2007; Zhang *et al.*, 2011). We attempted to synthesize a Zn^II^complex with the ligand in hydrothermal synthesis conditions. However the title compound was obtained, its structure is reported here.

The asymmetric unit comprises one 2-amino-5-(4-carboxylatophenyl)pyridinium molecule and one lattice water. The dihedral angel between the benzene ring and pyridinium ring is 6.63 (5)°, while the deprotonated carboxylate COO(O1—C12—O2) group is slightly twisted with an angle of 13.74 (3) ^o^ (Fig. 1). Intermolecular O—H···O and N—H···O hydrogen- bonding interactions (Table 1)) link adjacent molecules into a two-dimensional double layered supramolecular network (Fig. 2).

### Experimental

A mixture of 4-(6-aminopyridin-3-yl)benzoic acid (0.0214 g, 0.1 mmol), Zn(CH_3_COO)_2_.2H_2_O (0.0219 g, 0.1 mmol) and water (8 ml) was stired vigorously for 30 min and then sealed in a Teflon-lined stainless-steel autoclave. The autoclave was heated and maintained at 393 K for 2 days, and then cooled to room temperature at 5 K h^-1^ to obtain colorless prism crystals suitable for X-ray analysis.

### Refinement

The H atoms bonded to C and N atoms were positioned geometrically (C—H = 0.93 Å, N—H = 0.86 Å) and allowed to ride on their parent atoms, with *U*_iso_(H) value equal to 1.2*U*_eq_(C or N). The H atoms bonded to water O atoms were located in a difference Fourier map and refined with O—H distance restraint of 0.85±0.02 Å, *U*_iso_(H) = 1.5*U*_eq_(O).

### Crystal data

**Table:**

C_12_H_10_N_2_O_2_·H_2_O	*F*(000) = 488
*M**_r_* = 232.24	*D*_x_ = 1.377 Mg m^−^^3^
Monoclinic, *P*2_1_/*n*	Mo *K*α radiation, λ = 0.71073 Å
Hall symbol: P 2yn	Cell parameters from 1672 reflections
*a* = 7.796 (2) Å	θ = 2.8–27.5°
*b* = 7.808 (2) Å	µ = 0.10 mm^−^^1^
*c* = 18.480 (5) Å	*T* = 296 K
β = 95.165 (14)°	Prism, colorless
*V* = 1120.4 (6) Å^3^	0.23 × 0.16 × 0.15 mm
*Z* = 4	

### Data collection

**Table:**

Bruker SMART CCD diffractometer	2554 independent reflections
Radiation source: fine-focus sealed tube	1505 reflections with *I* > 2σ(*I*)
Graphite monochromator	*R*_int_ = 0.090
phi and ω scans	θ_max_ = 27.5°, θ_min_ = 2.8°
Absorption correction: multi-scan (*SADABS*; Sheldrick, 1996)	*h* = −10→10
*T*_min_ = 0.977, *T*_max_ = 0.985	*k* = −10→10
11449 measured reflections	*l* = −23→24

### Refinement

**Table:**

Refinement on *F*^2^	Primary atom site location: structure-invariant direct methods
Least-squares matrix: full	Secondary atom site location: difference Fourier map
*R*[*F*^2^ > 2σ(*F*^2^)] = 0.080	Hydrogen site location: inferred from neighbouring sites
*wR*(*F*^2^) = 0.208	H atoms treated by a mixture of independent and constrained refinement
*S* = 1.01	*w* = 1/[σ^2^(*F*_o_^2^) + (0.0766*P*)^2^ + 0.595*P*] where *P* = (*F*_o_^2^ + 2*F*_c_^2^)/3
2554 reflections	(Δ/σ)_max_ < 0.001
160 parameters	Δρ_max_ = 0.17 e Å^−^^3^
3 restraints	Δρ_min_ = −0.16 e Å^−^^3^

### Special details

**Table:**

Geometry. All e.s.d.'s (except the e.s.d. in the dihedral angle between two l.s. planes) are estimated using the full covariance matrix. The cell e.s.d.'s are taken into account individually in the estimation of e.s.d.'s in distances, angles and torsion angles; correlations between e.s.d.'s in cell parameters are only used when they are defined by crystal symmetry. An approximate (isotropic) treatment of cell e.s.d.'s is used for estimating e.s.d.'s involving l.s. planes.
Refinement. Refinement of *F*^2^ against ALL reflections. The weighted *R*-factor *wR* and goodness of fit *S* are based on *F*^2^, conventional *R*-factors *R* are based on *F*, with *F* set to zero for negative *F*^2^. The threshold expression of *F*^2^ > σ(*F*^2^) is used only for calculating *R*-factors(gt) *etc*. and is not relevant to the choice of reflections for refinement. *R*-factors based on *F*^2^ are statistically about twice as large as those based on *F*, and *R*- factors based on ALL data will be even larger.

### Fractional atomic coordinates and isotropic or equivalent isotropic displacement parameters (Å^2^)

**Table:**

	*x*	*y*	*z*	*U*_iso_*/*U*_eq_	
C1	0.5962 (4)	0.7159 (4)	0.15326 (17)	0.0568 (8)	
C2	0.6222 (4)	0.7885 (4)	0.08526 (18)	0.0669 (9)	
H2C	0.6003	0.9042	0.0767	0.080*	
C3	0.6796 (4)	0.6884 (4)	0.03225 (17)	0.0647 (9)	
H3A	0.6927	0.7373	−0.0128	0.078*	
C4	0.7203 (4)	0.5123 (4)	0.04277 (15)	0.0522 (7)	
C5	0.6949 (4)	0.4505 (4)	0.11010 (16)	0.0600 (8)	
H5A	0.7200	0.3362	0.1205	0.072*	
C6	0.7823 (4)	0.4004 (4)	−0.01377 (15)	0.0528 (7)	
C7	0.8241 (4)	0.4649 (4)	−0.08022 (16)	0.0642 (9)	
H7B	0.8121	0.5815	−0.0895	0.077*	
C8	0.8830 (4)	0.3594 (4)	−0.13264 (16)	0.0648 (9)	
H8C	0.9119	0.4067	−0.1761	0.078*	
C9	0.8999 (4)	0.1853 (4)	−0.12169 (15)	0.0555 (7)	
C10	0.8584 (5)	0.1196 (4)	−0.05641 (17)	0.0728 (10)	
H10A	0.8683	0.0025	−0.0478	0.087*	
C11	0.8022 (5)	0.2251 (4)	−0.00347 (18)	0.0770 (11)	
H11A	0.7770	0.1774	0.0404	0.092*	
C12	0.9664 (4)	0.0670 (4)	−0.17777 (17)	0.0634 (8)	
N1	0.6345 (3)	0.5496 (3)	0.16235 (13)	0.0606 (7)	
H1A	0.6200	0.5033	0.2036	0.073*	
N2	0.5351 (3)	0.8023 (4)	0.20782 (15)	0.0693 (8)	
H2A	0.5210	0.7511	0.2481	0.083*	
H2B	0.5100	0.9091	0.2027	0.083*	
O1	1.0359 (3)	0.1364 (3)	−0.22902 (12)	0.0757 (7)	
O2	0.9515 (4)	−0.0921 (3)	−0.17007 (13)	0.0853 (8)	
O3	0.4085 (4)	1.1412 (3)	0.16247 (15)	0.0866 (8)	
H3C	0.300 (3)	1.128 (6)	0.165 (2)	0.130*	
H3D	0.447 (5)	1.207 (5)	0.1977 (19)	0.130*	

### Atomic displacement parameters (Å^2^)

**Table:**

	*U*^11^	*U*^22^	*U*^33^	*U*^12^	*U*^13^	*U*^23^
C1	0.0564 (17)	0.0549 (18)	0.0606 (19)	−0.0067 (14)	0.0137 (14)	−0.0057 (14)
C2	0.085 (2)	0.0504 (17)	0.068 (2)	−0.0074 (16)	0.0206 (17)	0.0030 (16)
C3	0.082 (2)	0.0570 (18)	0.0581 (19)	−0.0106 (16)	0.0216 (16)	0.0042 (15)
C4	0.0583 (17)	0.0535 (17)	0.0454 (16)	−0.0112 (14)	0.0088 (13)	0.0015 (12)
C5	0.075 (2)	0.0542 (17)	0.0516 (17)	0.0010 (16)	0.0116 (15)	0.0009 (14)
C6	0.0579 (17)	0.0570 (17)	0.0441 (16)	−0.0110 (14)	0.0079 (13)	−0.0008 (13)
C7	0.091 (2)	0.0542 (17)	0.0480 (17)	−0.0022 (17)	0.0119 (16)	0.0042 (14)
C8	0.091 (2)	0.065 (2)	0.0397 (16)	−0.0048 (18)	0.0144 (15)	0.0046 (14)
C9	0.0617 (17)	0.0595 (18)	0.0452 (16)	−0.0059 (14)	0.0042 (13)	−0.0028 (13)
C10	0.109 (3)	0.058 (2)	0.0543 (19)	0.0010 (19)	0.0242 (18)	0.0045 (15)
C11	0.124 (3)	0.058 (2)	0.0527 (18)	−0.003 (2)	0.0330 (19)	0.0092 (15)
C12	0.072 (2)	0.069 (2)	0.0497 (18)	−0.0034 (17)	0.0087 (15)	−0.0035 (16)
N1	0.0786 (17)	0.0604 (16)	0.0444 (14)	−0.0014 (13)	0.0153 (12)	0.0001 (12)
N2	0.0809 (18)	0.0634 (16)	0.0668 (17)	−0.0017 (14)	0.0254 (15)	−0.0080 (13)
O1	0.1006 (18)	0.0747 (15)	0.0557 (13)	−0.0044 (13)	0.0287 (13)	−0.0042 (11)
O2	0.130 (2)	0.0615 (15)	0.0686 (16)	−0.0009 (15)	0.0326 (15)	−0.0012 (12)
O3	0.117 (2)	0.0677 (16)	0.0786 (18)	−0.0107 (16)	0.0272 (16)	−0.0095 (13)

### Geometric parameters (Å, º)

**Table:**

C1—N2	1.336 (4)	C8—C9	1.379 (4)
C1—N1	1.339 (4)	C8—H8C	0.9300
C1—C2	1.410 (4)	C9—C10	1.376 (4)
C2—C3	1.360 (4)	C9—C12	1.514 (4)
C2—H2C	0.9300	C10—C11	1.380 (4)
C3—C4	1.421 (4)	C10—H10A	0.9300
C3—H3A	0.9300	C11—H11A	0.9300
C4—C5	1.365 (4)	C12—O1	1.256 (4)
C4—C6	1.477 (4)	C12—O2	1.257 (4)
C5—N1	1.355 (4)	N1—H1A	0.8600
C5—H5A	0.9300	N2—H2A	0.8600
C6—C11	1.389 (4)	N2—H2B	0.8600
C6—C7	1.393 (4)	O3—H3C	0.860 (18)
C7—C8	1.381 (4)	O3—H3D	0.861 (18)
C7—H7B	0.9300		
			
N2—C1—N1	119.0 (3)	C9—C8—H8C	119.4
N2—C1—C2	124.0 (3)	C7—C8—H8C	119.4
N1—C1—C2	117.0 (3)	C10—C9—C8	118.0 (3)
C3—C2—C1	119.6 (3)	C10—C9—C12	119.6 (3)
C3—C2—H2C	120.2	C8—C9—C12	122.3 (3)
C1—C2—H2C	120.2	C9—C10—C11	120.9 (3)
C2—C3—C4	122.8 (3)	C9—C10—H10A	119.6
C2—C3—H3A	118.6	C11—C10—H10A	119.6
C4—C3—H3A	118.6	C10—C11—C6	122.0 (3)
C5—C4—C3	114.7 (3)	C10—C11—H11A	119.0
C5—C4—C6	121.3 (3)	C6—C11—H11A	119.0
C3—C4—C6	124.0 (3)	O1—C12—O2	124.3 (3)
N1—C5—C4	122.4 (3)	O1—C12—C9	116.8 (3)
N1—C5—H5A	118.8	O2—C12—C9	119.0 (3)
C4—C5—H5A	118.8	C1—N1—C5	123.5 (3)
C11—C6—C7	116.4 (3)	C1—N1—H1A	118.2
C11—C6—C4	121.7 (3)	C5—N1—H1A	118.2
C7—C6—C4	121.8 (3)	C1—N2—H2A	120.0
C8—C7—C6	121.4 (3)	C1—N2—H2B	120.0
C8—C7—H7B	119.3	H2A—N2—H2B	120.0
C6—C7—H7B	119.3	H3C—O3—H3D	108 (3)
C9—C8—C7	121.2 (3)		
			
N2—C1—C2—C3	−177.8 (3)	C7—C8—C9—C10	−0.8 (5)
N1—C1—C2—C3	1.7 (5)	C7—C8—C9—C12	−179.2 (3)
C1—C2—C3—C4	−2.0 (5)	C8—C9—C10—C11	−0.3 (5)
C2—C3—C4—C5	0.9 (5)	C12—C9—C10—C11	178.1 (3)
C2—C3—C4—C6	179.8 (3)	C9—C10—C11—C6	1.1 (6)
C3—C4—C5—N1	0.4 (4)	C7—C6—C11—C10	−0.8 (5)
C6—C4—C5—N1	−178.5 (3)	C4—C6—C11—C10	179.3 (3)
C5—C4—C6—C11	6.1 (5)	C10—C9—C12—O1	−165.4 (3)
C3—C4—C6—C11	−172.8 (3)	C8—C9—C12—O1	12.9 (5)
C5—C4—C6—C7	−173.9 (3)	C10—C9—C12—O2	13.6 (5)
C3—C4—C6—C7	7.3 (5)	C8—C9—C12—O2	−168.1 (3)
C11—C6—C7—C8	−0.3 (5)	N2—C1—N1—C5	179.1 (3)
C4—C6—C7—C8	179.6 (3)	C2—C1—N1—C5	−0.4 (5)
C6—C7—C8—C9	1.2 (5)	C4—C5—N1—C1	−0.7 (5)

### Hydrogen-bond geometry (Å, º)

**Table:**

*D*—H···*A*	*D*—H	H···*A*	*D*···*A*	*D*—H···*A*
N1—H1*A*···O1^i^	0.86	1.82	2.648 (3)	160
N2—H2*A*···O2^i^	0.86	2.07	2.911 (4)	167
N2—H2*B*···O3	0.86	2.09	2.921 (4)	163
O3—H3*C*···O2^ii^	0.86 (2)	1.99 (2)	2.849 (4)	178 (4)
O3—H3*D*···O1^iii^	0.86 (2)	1.91 (2)	2.768 (4)	176 (5)

Symmetry codes: (i) *x*−1/2, −*y*+1/2, *z*+1/2; (ii) −*x*+1, −*y*+1, −*z*; (iii) *x*−1/2, −*y*+3/2, *z*+1/2.
